# Synthesis and cellular bioactivities of novel isoxazole derivatives incorporating an arylpiperazine moiety as anticancer agents

**DOI:** 10.1080/14756366.2018.1504041

**Published:** 2018-09-24

**Authors:** Burcu Çalışkan, Esra Sinoplu, Kübra İbiş, Ece Akhan Güzelcan, Rengül Çetin Atalay, Erden Banoglu

**Affiliations:** a Department of Pharmaceutical Chemistry, Faculty of Pharmacy, Gazi University, Ankara, Turkey;; b Department of Bioinformatics, Middle East Technical University, Ankara, Turkey

**Keywords:** Isoxazole, piperazine, liver cancer, oxidative stress, cytotoxicity

## Abstract

In our endeavour towards the development of effective anticancer therapeutics, a novel series of isoxazole-piperazine hybrids were synthesized and evaluated for their cytotoxic activities against human liver (Huh7 and Mahlavu) and breast (MCF-7) cancer cell lines. Within series, compounds **5l**-**o** showed the most potent cytotoxicity on all cell lines with IC_50_ values in the range of 0.3–3.7 μM. To explore the mechanistic aspects fundamental to the observed activity, further biological studies with **5m** and **5o** in liver cancer cells were carried out. We have demonstrated that **5m** and **5o** induce oxidative stress in PTEN adequate Huh7 and PTEN deficient Mahlavu human liver cancer cells leading to apoptosis and cell cycle arrest at different phases. Further analysis of the proteins involved in apoptosis and cell cycle revealed that **5m** and **5o** caused an inhibition of cell survival pathway through Akt hyperphosphorylation and apoptosis and cell cycle arrest through p53 protein activation.

## Introduction

Cancer is one of the leading cause of deaths globally, and can be classified as a multifactorial disease, which is diligently orchestrated by a combination of genetic, epigenetic, and environmental factors working together towards the progression of tumours^1^^,^^2^. Hepatocellular carcinoma (HCC) is the most common type of liver cancer accounting for about 75% of all primary liver cancers and is the 6th most frequent and the 2nd deadly cancer worldwide^3^. Etiological factors, which are associated with HCC are chronic infection by hepatitis B virus (HBV) and hepatitis C virus (HCV), alcohol consumption, obesity, and aflatoxin exposure^4^^,^^5^. Primary liver cancer is extremely resistant to conventional chemotherapeutics, only 7% of patients have five-year survival^6^. Sorafenib and Regorafenib are the only FDA approved agents for advanced liver cancer cases, which extend patient survival approximately 3–7 months^7^^,^^8^. One of the biggest challenge in anticancer drug development is that HCC cells are reported to display high levels of cellular heterogeneity^9^, which hampers significantly the development of new cancer therapeutics and causes failures in clinical studies since many candidate drugs do not produce clinical benefit in the overall population^10^. Consequently, an endless effort has continuously been devoted to the discovery and development of new and more effective anticancer agents that are capable to intervene with this complex disease.

Diverse azaheterocyclic ring systems have been in the centre of medicinal chemists, and used as versatile tools and building blocks for the synthesis of small molecule cancer therapeutics^11^. Among them, one of the most constantly growing area was the investigation of the antitumor properties of compounds containing isoxazole core structure^12^. For example, a series of resorcinylic 4,5-diarylisoxazole amides have been developed as potent heat shock protein (HSP90) inhibitors, exemplified by NVP-AUY922 (Luminespib)^13^, which was active against a variety of tumor xenografts and has been evaluated in phase II clinical trials^14^. Currently marketed immunosuppresory drug Leflunomide, which has recently been identified as a potential anticancer drug^15^, is also an isoxazole derivative. Isoxazole derivatives as comberastatin A-4 analogues are also successfully described as tubulin polymerisation inhibitors with antiproliferative activities towards various cell lines^16^^,^^17^. Hewings and others reported the 3,5-dimethylisoxazole moiety as an effective acetylated lysine (KAc) mimic, which was used for developing bromodomain inhibitors with anticancer activity^18–21^. In addition, a naturally occurring diarylisoxazole derivative has recently been reported as a new chemical tool with efficacy against AR-expressing breast cancer cells^22^.

Another widely occurring structural fragment in anticancer compounds is the piperazine moiety and a large number of compounds have appeared in the literature having piperazine motif with cytotoxicity against various cancer cells^23–27^. For instance, studies on an arylpiperazine derivative naftopidil^28^^,^^29^, a well-known α_1_-adrenergic receptor antagonist, and several other arylpiperazines have shown significant cytotoxicity against prostate cancer cells^30–32^. A series of piperazine derivatives have also been demonstrated to bear potent antiproliferative activities against various cancer cells including colon, prostate, breast, lung, and leukaemia as well as to suppress experimental tumours in small animal models by a mechanism involving inhibition of microtubule synthesis, inhibition of cell-cycle progression and angiogenesis^33^^,^^34^. Recently, incorporation of arylpiperazine moiety in purine nucleoside analogues resulted in compounds with senescence-induced cell death in liver cancer cells^26^. Other recent progress on anticancer piperazine derivatives can be found elsewhere^35^.

In the course of our ongoing research interest concerning bioactive heterocycles^36–40^, we have relied on the aforementioned data for the design, synthesis, and biological evaluation of novel isoxazole derivatives containing in their structural framework an aryl piperazine residue. Since primary liver cancer incidence is expected to increase due to the obesity associated non-alcoholic fatty liver disease^4^, we think that the synthesis of novel anticancer agents as reported herein will contribute not only the mechanistic bioactivity analysis of these isoxazole-piperazine hybrids, but also the future treatment options for HCC. In this context, we hereby report the straightforward synthesis of the novel isoxazole-piperazine hybrids, which were evaluated for their antitumor activities.

## Experimental

### Chemistry

Starting materials were purchased from commercial suppliers and used without further purification. ^1^H and ^13^C NMR spectra were recorded in CDCl_3_ or DMSO-d_6_ on a Varian Mercury 400 MHz spectrometer (Agilent Technologies, Santa Clara, CA, USA) using tetramethylsilane as the internal standard. All chemical shifts were recorded as δ (ppm), coupling constants are reported as Hertz. High resolution mass spectra data (HRMS) were collected using Waters LCT Premier XE Mass Spectrometer (high sensitivity orthogonal acceleration time-of-flight instrument) operating in ESI (+) or ESI (−) method, also coupled to an AQUITY Ultra Performance Liquid Chromatography system (Waters Corporation, Milford, MA, USA) using a UV detector monitoring at 254 nm. Purity for all final compounds were >95%, according to the UPLC-MS method using (A) water + 0.1% formic acid and (B) acetonitrile + 0.1% Formic Acid; flow rate = 0.3 mL/min, Column: Aquity BEH C18 column (2.1 × 100 mm, 1.7 mm; Waters Corporation, Milford, MA, USA). Flash chromatography on silica gel was performed on RediSep prepacked disposable silica gel columns using Teledyne Isco Combiflash. Preparative liquid chromatography was performed on Vydac Denali C18 Column (150 × 20 mm, 5 µ; Grace, Columbia, MD, USA) using Reveleris PREP purification system. All microwave irradiation experiments were carried out in a Biotage Initiator + microwave apparatus with Biotage sealed microvawe vials. Melting points of the synthesized compounds were determined by SMP50 automatic melting point apparatus and uncorrected. Experimental data for all intermediate compounds can be found in Supporting Information.

### Synthesis of Compounds 5a-o

The mixture of the appropriate bromide derivative (**4a-o**) (0.5 mmol, 1 eq), 4-trifluoromethylbenzylpiperazine (0.6 mmol, 1.2 eq) and DIEA (1 mmol, 2 eq) in DMF (2 ml) was heated by microwave irradiation at 80 °C for 20 min. Then, it was poured into ice-water and formed precipitate was filtrated. The crude product was purified by flash chromatography.

#### 5-Phenyl-3-((4-(4-(trifluoromethyl)benzyl)piperazin-1-yl)methyl)isoxazole (5a)

Purified by flash column chromatography (0% → 10% MeOH in DCM). Yield 87.0%; mp 112.6–113.9 °C. ^1^H NMR (CDCl_3_): δ 2.51 (4H, bs), 2.59 (4H, bs), 3.57 (2H, s), 3.66 (2H, s), 6.55 (1H, s), 7.42–7.48 (5H, m), 7.56 (2H, d, *J* = 8.0 Hz), 7.76–7.78 (2H, m). ^13^C NMR (CDCl_3_) δ 52.88, 52.98, 53.29, 62.26, 99.60, 124.20 (q, ^1^*J_C-F_* = 270.0 Hz), 125.15 (q, ^3^*J_C-F_* = 3.9 Hz), 125.77, 127.51, 128.92, 129.18, 129.44 (q, ^2^*J_C-F_* = 30.0 Hz), 130.08, 142.26, 161.68, 169.97. HRMS (*m/z*) [M + H]^+^ calcd for C_22_H_23_F_3_N_3_O: 402.1793, found, 402.1794.

#### 5-(4-Fluorophenyl)-3-((4-(4-(trifluoromethyl)benzyl)piperazin-1-yl)methyl)isoxazole (5b)

Purified by flash column chromatography (0% → 10% MeOH in DCM). Yield 74.0%; mp 110.1–111.0 °C. ^1^H NMR (CDCl_3_): δ 2.60–2.72 (8H, m), 3.64 (2H, s), 3.72 (2H, s), 6.58 (1H, s), 7.12–7.18 (2H, m), 7.49–7.52 (2H, m), 7.58 (2H, d, *J* = 8.4 Hz), 7.74–7.79 (2H, m). ^13^C NMR (CDCl_3_) δ 53.06, 53.19, 53.46, 62.47, 99.60, 116.37 (d, ^2^*J_C-F_* = 21.8 Hz), 124.05 (d, ^4^*J_C-F_* = 3.2 Hz), 124.40 (q, ^1^*J_C-F_* = 270.5 Hz), 125.38 (q, ^3^*J_C-F_* = 3.8 Hz), 128.04 (d, ^3^*J_C-F_* = 8.4 Hz), 129.41, 142.46, 162.05, 163.90 (d, ^1^*J_C-F_* = 250.0 Hz), 169.20. HRMS (*m/z*) [M + H]^+^ calcd for C_22_H_22_F_4_N_3_O: 420.1699, found, 420.1686.

#### 5-(4-Chlorophenyl)-3-((4-(4-(trifluoromethyl)benzyl)piperazin-1-yl)methyl)isoxazole (5c)

Purified by flash column chromatography (0% → 20% EA in DCM). Yield 68.1%; mp 126.2–126.9 °C. ^1^H NMR (CDCl_3_): δ 2.52 (4H, bs), 2.59 (4H, bs), 3.57 (2H, s), 3.66 (2H, s), 6.55 (1H, s), 7.41–7.45 (4H, m), 7.56 (2H, d, *J* = 7.6 Hz), 7.69 (2H, d, *J* = 8.4 Hz). ^13^C NMR (CDCl_3_) δ 52.80, 52.94, 53.21, 62.23, 99.95, 124.19 (q, ^1^*J_C-F_* = 270.6 Hz), 125.15 (q, ^3^*J_C-F_* = 3.8 Hz), 125.91, 127.03, 129.24, 129.28, 136.18, 141.95, 161.79, 168.86. HRMS (*m/z*) [M + H]^+^ calcd for C_22_H_22_ClF_3_N_3_O: 436.1404, found, 436.1402.

#### 5-(p-Tolyl)-3-((4-(4-(trifluoromethyl)benzyl)piperazin-1-yl)methyl)-isoxazole (5d)

Purified by flash column chromatography (0% → 10% MeOH in DCM). Yield 84.0%; mp 141.0–142.3 °C. ^1^H NMR (CDCl_3_): δ 2.40 (3H. s), 2.61 (4H, bs), 2.69 (4H, bs), 3.63 (2H, s), 3.71 (2H, s), 6.57 (1H, s), 7.27 (2H, d, *J* = 8.0 Hz), 7.48 (2H, d, *J* = 7.6 Hz), 7.58 (2H, d, *J* = 7.6 Hz), 7.66 (2H, d, *J* = 8.0 Hz). ^13^C NMR (CDCl_3_) δ 21.66, 53.07, 53.17, 53.52, 62.48, 99.22, 124.41 (q, ^1^*J_C-F_* = 269.8 Hz), 124.98, 125.37 (q, ^3^*J_C-F_* = 3.8 Hz), 125.91, 129.41, 129.82, 140.60, 142.50, 161.81, 170.36. HRMS (*m/z*) [M + H]^+^ calcd for C_23_H_25_F_3_N_3_O: 416.1950, found, 416.1948.

#### 3-((4-(4-(Trifluoromethyl)benzyl)piperazin-1-yl)methyl)-5-(4-(trifluoromethyl) phenyl)isoxazole (5e)

Purified by flash column chromatography (0% → 10% MeOH in DCM). Yield 44.0%; mp 110.1–111.5 °C. ^1^H NMR (CDCl_3_): δ 2.53 (4H, bs), 2.61 (4H, bs), 3.58 (2H, s), 3.68 (2H, s), 6.67 (1H, s), 7.45 (2H, d, *J* = 8.2 Hz), 7.56 (2H, d, *J* = 8.2 Hz), 7.72 (2H, d, *J* = 7.8 Hz), 7.88 (2H, d, *J* = 7.8 Hz). ^13^C NMR (CDCl_3_) δ 52.83, 52.99, 53.19, 62.23, 101.08, 123.71(q, ^1^*J_C-F_* = 270.6 Hz), 124.19 (q, ^1^*J_C-F_* = 270.5 Hz), 125.17 (q, ^3^*J_C-F_* = 3.9 Hz), 125.99 (q, ^3^*J_C-F_* = 3.8 Hz), 126.03, 129.19, 129.50 (q, ^2^*J_C-F_* = 31.0 Hz), 130.58, 131.84 (q, ^2^*J_C-F_* = 32.7 Hz), 142.18, 162.01, 168.33. HRMS (*m/z*) [M + H]^+^ calcd for C_23_H_22_F_6_N_3_O: 470.1667, found, 470.1667.

#### 5-(4-Isopropylphenyl)-3-((4-(4-(trifluoromethyl)benzyl)piperazin-1-yl)methyl) isoxazole (5f)

Purified by flash column chromatography (0% → 10% MeOH in DCM). Yield 67.0%; mp 111.8–112.2 °C. ^1^H NMR (CDCl_3_): δ 1.27 (6H, d, *J* = 6.8 Hz), 2.51 (4H, bs), 2.58 (4H, bs), 2.93–2.96 (1H, m), 3.57 (2H, s), 3.65 (2H, s), 6.51 (1H, s), 7.31 (2H, d, *J* = 8.2 Hz), 7.44 (2H, d, *J* = 7.8 Hz), 7.56 (2H, d, *J* = 7.8 Hz), 7.69 (2H, d, *J* = 8.2 Hz). ^13^C NMR (CDCl_3_) δ 23.76, 34.08, 52.83, 52.94, 53.30, 62.25, 99.06, 124.21 (q, ^1^*J_C-F_* = 270.0 Hz), 125.10, 125.18 (q, ^3^*J_C-F_* = 3.8 Hz), 125.84, 127.04, 129.23, 129.40 (q, ^2^*J_C-F_* = 34.0 Hz), 142.15, 151.30, 161.53, 170.19. HRMS (*m/z*) [M + H]^+^ calcd for C_25_H_29_F_3_N_3_O: 444.2263, found, 444.2265.

#### 5-(4-(Trifluoromethoxy)phenyl)-3-((4-(4-(trifluoromethyl)benzyl)piperazin-1-yl)methyl)isoxazole (5g)

Purified by flash column chromatography (0% → 10% MeOH in DCM). Yield 85.0%; mp 103.2–103.9 °C. ^1^H NMR (CDCl_3_): δ 2.59 (4H, bs), 2.67 (4H, bs), 3.63 (2H, s), 3.71 (2H, s), 6.62 (1H, s), 7.31 (2H, d, *J* = 8.4 Hz), 7.47 (2H, d, *J* = 8.0 Hz), 7.58 (2H, d, *J* = 8.0 Hz), 7.81 (2H, d, *J* = 8.4 Hz). ^13^C NMR (CDCl_3_) δ 53.05, 53.21, 53.44, 62.46, 100.30, 120.55 (q, ^1^*J_C-F_* = 257.0 Hz), 121.53, 124.40 (q, ^1^*J_C-F_* = 270.5 Hz), 125.39 (q, ^3^*J_C-F_* = 3.8 Hz), 126.28, 127.61, 129.41, 142.50, 150.50, 162.15, 168.76. HRMS (*m/z*) [M + H]^+^ calcd for C_23_H_22_F_6_N_3_O_2_: 486.1616, found, 486.1600.

#### 5-(4-(Methylsulfonyl)phenyl)-3-((4-(4-(trifluoromethyl)benzyl)piperazin-1-yl)methyl)isoxazole (5h)

Purified by flash column chromatography (0% → 10% MeOH in DCM). Yield 84.0%; mp 163.5–164.1 °C. ^1^H NMR (CDCl_3_): δ 2.51 (4H, bs), 2.59 (4H, bs), 3.08 (3H, s), 3.57 (2H, s), 3.68 (2H, s), 6.73 (1H, s), 7.44 (2H, d, *J* = 7.8 Hz), 7.56 (2H, d, *J* = 7.8 Hz), 7.96 (2H, d, *J* = 8.8 Hz), 8.04 (2H, d, *J* = 8.8 Hz). ^13^C NMR (CDCl_3_) δ 44.43, 52.83, 53.00, 53.17, 62.24, 101.94, 124.19 (q, ^1^*J_C-F_* = 269.8 Hz), 125.20 (q, ^3^*J_C-F_* = 3.6 Hz), 126.52, 128.20, 129.23, 132.11, 141.55, 142.09, 162.21, 167.69. HRMS (*m/z*) [M + H]^+^ calcd for C_23_H_25_F_3_N_3_O_3_S: 480.1569, found, 480.1567.

#### 4-(3-((4-(4-(Trifluoromethyl)benzyl)piperazin-1-yl)methyl)isoxazol-5-yl)phenol (5i)

Purified by flash column chromatography (0% → 10% MeOH in DCM). Yield 71.6%; mp 154.2–156.0 °C. ^1^H NMR (CDCl_3_): δ 2.56 (4H, bs), 2.66 (4H, bs), 3.57 (2H, s), 3.67 (2H, s), 6.22 (1H, s), 6.70 (2H, d, *J* = 8.4 Hz), 7.42–7.47 (4H, m), 7.56 (2H, d, *J* = 8.4 Hz). ^13^C NMR (CDCl_3_): δ 52.83, 53.18, 62.47, 98.64, 116.31, 119.78, 124.20 (q, ^1^*J_C-F_* = 270.0 Hz), 125.46 (q, ^3^*J_C-F_* = 3.1 Hz), 127.65, 129.62, 142.10, 158.19, 160.44, 170.43. HRMS (*m/z*) [M + H]^+^ calcd for C_22_H_23_F_3_N_3_O_2_: 418.1742; found, 418.1736.

#### 5-(4-Methoxyphenyl)-3-((4-(4-(trifluoromethyl)benzyl)piperazin-1-yl)methyl)isoxazole (5j)

Purified by flash column chromatography (0% → 10% MeOH in DCM). Yield 59.4%; mp 117.9–118.2 °C. ^1^H NMR (CDCl_3_): δ 2.49 (4H, bs), 2.57 (4H, bs), 3.55 (2H, s), 3.63 (2H, s), 3.85 (3H, s), 6.42 (1H, s), 6.96 (2H, d, *J* = 9.2 Hz), 7.43 (2H, d, *J* = 7.8 Hz), 7.55 (2H, d, *J* = 7.8 Hz), 7.70 (2H, d, *J* = 9.2 Hz). ^13^C NMR (CDCl_3_) δ 52.94, 53.05, 53.37, 55.37, 62.33, 98.24, 114.34, 120.35, 124.23 (q, ^1^*J_C-F_* = 270.0 Hz), 125.14 (q, ^3^*J_C-F_* = 3.8 Hz), 127.35, 129.17, 129.32 (q, ^2^*J_C-F_* = 32.0 Hz), 142.40, 161.03, 161.72, 169.90. HRMS (*m/z*) [M + H]^+^ calcd for C_23_H_25_F_3_N_3_O_2_: 432.1899, found, 432.1891.

#### 5-(4-Propoxyphenyl)-3-((4-(4-(trifluoromethyl)benzyl)piperazin-1-yl)methyl)isoxazole (5k)

Purified by flash column chromatography (0% → 10% MeOH in DCM). Yield 79.0%; mp 103.0–103.5 °C. ^1^H NMR (CDCl_3_): δ 1.05 (3H, t, *J* = 7.4 Hz), 1.80–1.85 (2H, m), 2.51 (4H, bs), 2.58 (4H, bs), 3.56 (2H, s), 3.64 (2H, s), 3.96 (2H, t, *J* = 6.4 Hz), 6.42 (1H, s), 6.95 (2H, d, *J* = 8.8 Hz), 7.43 (2H, d, *J* = 7.8 Hz), 7.56 (2H, d, *J* = 7.8 Hz), 7.68 (2H, d, *J* = 8.8 Hz). ^13^C NMR (CDCl_3_) δ 10.46, 22.48, 52.82, 52.92, 53.30, 62.25, 69.64, 98.17, 114.85, 120.07, 124.21 (q, ^1^*J_C-F_* = 270.6 Hz), 125.18 (q, ^3^*J_C-F_* = 3.8 Hz), 127.33, 129.24, 129.40 (q, ^2^*J_C-F_* = 32.7 Hz), 142.10, 160.66, 161.48, 170.07. HRMS (*m/z*) [M + H]^+^ calcd for C_25_H_29_F_3_N_3_O_2_: 460.2212, found, 460.2213.

#### 5-(4-(Allyloxy)phenyl)-3-((4-(4-(trifluoromethyl)benzyl)piperazin-1-yl)methyl)isoxazole (5l)

Purified by flash column chromatography (0% → 10% MeOH in DCM). Yield 78.5%; mp 107.1–107.4 °C. ^1^H NMR (CDCl_3_): δ 2.51 (4H, bs), 2.58 (4H, bs), 3.56 (2H, s), 3.64 (2H, s), 4.58 (2H, dt, *J* = 5.6 Hz, 1.6 Hz), 5.33 (1H, dq, *J* = 10.4, 1.4 Hz), 5.44 (1H, dq, *J* = 17.2, 1.6 Hz), 6.01-6.10 (1H, m), 6.43 (1H, s), 6.97 (2H, d, *J* = 9.0 Hz), 7.44 (2H, d, *J* = 7.8 Hz), 7.56 (2H, d, *J* = 7.8 Hz), 7.69 (2H, d, *J* = 9.0 Hz). ^13^C NMR (CDCl_3_) δ 52.66, 52.78, 53.20, 62.14, 68.86, 98.35, 115.12, 118.05, 120.38, 124.18 (q, ^1^*J_C-F_* = 270.6 Hz), 125.23 (q, ^3^*J_C-F_* = 3.8 Hz), 127.36, 129.32, 132.70, 142.02, 160.07, 161.25, 170.04. HRMS (*m/z*) [M + H]^+^ calcd for C_25_H_27_F_3_N_3_O_2_: 458.2055, found, 458.2061.

#### 5-(4-((3-Methylbut-2-en-1-yl)oxy)phenyl)-3-((4-(4-(trifluoromethyl)benzyl)piperazin-1-yl)methyl)isoxazole (5m)

Purified by flash column chromatography (0% → 10% MeOH in DCM). Yield 66.8%; mp 100.0–100.3 °C. ^1^H NMR (CDCl_3_): δ 1.76 (3H, s), 1.81 (3H, s), 2.53 (4H, bs), 2.60 (4H, bs), 3.58 (2H, s), 3.66 (2H, s), 4.55 (2H, d, *J* = 6.8 Hz), 5.47–5.51 (1H, m), 6.44 (1H, s), 6.97 (2H, d, *J* = 8.8 Hz), 7.45 (2H, d, *J* = 8.0 Hz), 7.56 (2H, d, *J* = 8.0 Hz), 7.69 (2H, d, *J* = 8.8 Hz). ^13^C NMR (CDCl_3_) δ 18.23, 25.81, 52.86, 52.95, 53.31, 62.26, 64.93, 98.21, 115.05, 119.15, 120.18, 124.10 (q, ^1^*J_C-F_* = 270.6 Hz), 125.18 (q, ^3^*J_C-F_* = 3.2 Hz), 127.33, 129.23, 138.72, 160.38, 170.04. HRMS (*m/z*) [M + H]^+^ calcd for C_27_H_31_F_3_N_3_O_2_: 486.2368, found, 486.2362.

#### 3,5-Dimethyl-4-((4-(3-((4-(4-(trifluoromethyl)benzyl)piperazin-1-yl)methyl)isoxazol-5-yl)phenoxy)methyl)isoxazole hydrochloride (5n)

Purified by flash column chromatography (0% → 10% MeOH in DCM). Yield 58.4%; mp 200.4–201.2 °C (decomp). ^1^H NMR (DMSO-d_6_): δ 2.23 (3H, s), 2.28 (3H, s), 2.43 (3H, s), 3.40 (8H, bs), 4.33 (2H, s), 4.47 (2H, s), 5.02 (2H, s), 7.09 (1H, s), 7.19 (2H, d, *J* = 8.8 Hz), 7.80–7.85 (4H, m), 7.90 (2H, d, *J* = 8.0 Hz). ^13^C NMR (DMSO-d_6_) δ 9.70, 10.65, 47.99, 48.01, 49.68, 57.38, 59.23, 100.13, 110.08, 115.70, 119.50, 124.00 (q, ^1^*J_C-F_* = 243.6 Hz), 125.59 (q, ^3^*J_C-F_* = 3.8 Hz), 127.36, 129.61, 130.08 (q, ^2^*J_C-F_* = 32.1 Hz), 132.25, 159.54, 159.92, 167.60, 169.81. HRMS (*m/z*) [M + H]^+^ calcd for C_28_H_30_ClF_3_N_4_O_3_: 527.2270, found, 527.2275.

#### 5-(4-((1,3-Dimethyl-1H-pyrazol-4-yl)methoxy)phenyl)-3-((4-(4-(trifluoromethyl)benzyl)piperazin-1-yl)methyl)isoxazole (5o)

Purified by flash column chromatography (0% → 10% MeOH in DCM). Yield 58.0%; mp 131.4–131.9 °C. ^1^H NMR (CDCl_3_): δ 2.52 (3H, s), 2.51 (4H, bs), 2.58 (4H, bs), 3.57 (2H, s), 3.64 (2H, s), 3.84 (3H, s), 5.03 (2H, s), 6.11 (1H, s), 6.45 (1H, s), 7.02 (2H, d, *J* = 8.8 Hz), 7.44 (2H, d, *J* = 7.8 Hz), 7.56 (2H, d, *J* = 7.8 Hz), 7.71 (2H, d, *J* = 8.8 Hz). ^13^C NMR (CDCl_3_) δ 13.38, 36.46, 52.40, 52.56, 53.05, 60.62, 61.98, 98.71, 106.88, 115.18, 120.99, 124.13 (q, ^1^*J_C-F_* = 270.6 Hz), 125.31 (q, ^3^*J_C-F_* = 3.8 Hz), 127.50, 129.45, 137.26, 147.38, 159.50, 160.95, 169.91. HRMS (*m/z*) [M + H]^+^ calcd for C_28_H_31_F_3_N_5_O_2_: 526.2430, found, 526.2429.

### Biology

#### Cell culture

Huh7 (epithelial-like) and Mahlavu (mesenchymal-like) human hepatocellular cancer cell lines and MCF7 human breast cancer carcinoma cells were grown in Dulbecco’s Modified Eagles Medium(DMEM) supplemented with %10 fetal bovine serum (Gibco, Invitrogen, Carlsbad, CA, USA), 1% non-essential amino acids (Gibco, Invitrogen) and 100 units/ml penicillin/streptomycin (Gibco, Invitrogen). Cells were maintained at 37 °C in a humidified incubator under 5% CO_2._

#### NCI-60 sulforhodamine B assay

Huh7, MCF7 (2500 cell/well in 150 µl/well) and Mahlavu (1000 cell/well in 150 µl/well) cells were plated in 96-well plates and were grown in incubator for 24 hours. The compounds were dissolved in DMSO (Sigma, St Louis, MO, USA) as 20 mM stock solution. The compounds which were below 2.5 µM were tested in a concentration range of starting from 2.5 µM to 0.015 µM. Cells were fixed using 10% (*v/v*) trichloroacetic acid (Sigma ) for an hour after the end of 72 h incubation time. The fixed plates were dried and fixed cells were stained with sulforhodamine B (SRB) solution (Sigma) (50 µl of a 0.4% (*m/v*) of SRB in 1% acetic acid solution (Sigma)) for 10 min. In order to remove unbound SRB dye, cells were washed with 1% acetic acid three times and left for air-drying. The protein bound SRB dye was dissolved in 10 mM Tris-base (Sigma) and absorbance was measured with 96-well plate reader at 515 nm. The IC_50_ values were calculated and the cells treated with DMSO alone were used as control. All experiments were done in triplicate. Data with *R*^2^ values >0.9 was considered significant.

#### Real-time cell growth surveillance by cell electronic sensing (RT-CES)

Real-time cell growth analysis was performed using the xCELLigence System (Roche Applied Sciences, Penzberg, Germany). The Huh7, MCF7 (2500 cell/well) and Mahlavu (1000 cell/well) cells were seeded in E-Plates 96. In proliferation step, the cellular growth was analysed with cell index measurements in every 30 min for 24 h. After 24 h of incubation, when cells reached the log growth phase, they were treated with **5m** and **5o** starting from 10 µM and 1/2 folds’ serial dilutions three times. The cell index values (CI) were initially monitored every 10 min for 24 h and then CI were recorded in 30 min intervals. After 72 h of incubation, the cellular growth ratios were calculated by CI_drug_/CI_DMSO_.

#### Oxidative stress assay

Mahlavu (35000 cells/well) and Huh7 (50000 cells/well) cells were inoculated into 6-wells plate for 24 h. Then cells were treated with **5o** (1 µM for Huh7 and 4 µM for Mahlavu) and **5m** (1 µM for Huh7 and Mahlavu). One group of cells did not receive the compounds, but they were grown in selenium-deficient serum-free medium as positive control for oxidative stress^41^. Fourth group was treated with DMSO as negative control. At the end of 24 h, 48 h, and 72 h incubation period, the cells were collected and analysed by MUSE Oxidative Stress Kit (MCH100111, Merck Millipore, Burlington, MA, USA), which uses dihydroethium to monitor superoxide production in the cells^42^. Compound **5o** impaired Mahlavu cells a lot, so 10,000 events were analysed for treated cells, 2000 events could be done in **5o** treated Mahlavu cells. In parallel, Huh7 cells were plated into six-well plates for 24 h followed by treatment with **5o** (1 µM) or **5m** (1 µM) or DMSO or selenium deficient serum-free medium. After 24 h, 48 h, 72 h treatment periods, samples were washed three times with 1 × PBS, then they were incubated with dichloro-dihydro-fluorescein diacetate (DCFH-DA) solution (10 mM glucose, 0.5 µM DCFH-DA, 10 mM HEPES dissolved in 1 × PBS) in order to detect ROS (particularly H_2_O_2_) in the cells for 15 min in humidified chamber in dark at 37 °C. The solution was aspirated and cells were washed with PBS two times. The staining was analysed *in situ* with fluorescence microscope.

#### Flow cytometry for cell cycle analysis

Huh7 and Mahlavu cells were seeded onto 100 mm culture dishes. After 24 h, cells were treated with **5o** (1 µM for Huh7 and 4 µM for Mahlavu) or **5m** (1 µM for Huh7 and Mahlavu) or DMSO as a negative control. The end of 24 h, 48 h, and 72 h of incubation period, cells were fixed with ice-cold 70% ethanol for 3 h at −20 °C. Cell cycle analysis was carried out by PI (propidium iodide) staining using MUSE Cell Analyzer according to the manufacturer’s recommendations (Millipore).

#### Immunofluorescence staining

Huh7 (50,000 cells/well) and Mahlavu (35,000 cells/well) cells were inoculated on cover slides in 6-well plates after 24 h, cells were treated with **5o** (1 µM for Huh7 and 4 µM for Mahlavu) or **5m** (1 µM for Huh7 and Mahlavu) or DMSO control for 24 h, 48 h, and 72 h. After incubation time periods, the cells were washed three times with 1 × PBS and fixed with %100 ice-cold methanol. Then, the cells were stained with 1 µg/ml Hoechst (#33258, Sigma). Finally, the cells were analysed under a fluorescent microscope.

#### Western blot analysis

Cells were treated with the **5o** (1 µM for Huh7 and 4 µM for Mahlavu), **5m** (1 µM for Huh7 and Mahlavu) and with DMSO as control for 72 h. After 72 h incubation, the cells were collected with scraper, their total proteins were isolated and protein concentrations were calculated with Bradford assay. Bio-Rad protein electrophoresis (Mini-PROTEAN^®^ TetraCellSystems and TGX™ precast gels, Bio-Rad, Hercules, CA, USA) and transfer system (Trans-Blot^®^ TurboTransfer System, Bio-Rad, Hercules, CA, USA) were used according to the manufacturer’s protocol for all the Western blotting analyses. About 20–40 µg of protein were used per well. Proteins were transferred to a PVDF membrane. For immunoblotting, PARP (#9532S, Cell Signaling), p21/WAF1/Cip1 (#05-345, Millipore), p53 (#05-224, Millipore), phospho-p53^Ser15^ (#9286S, Cell Signaling), Rb (#9309, Cell Signaling), and phospho-Rb^Ser807/811^ (#9308S, Cell Signaling), α-phospho-Akt^Ser473^ (Cell Signaling, #9271), and AKT (#9272, Cell Signaling) antibodies were used in 1:100 to 1:500 5% BSA-TBS-T. β-actin (#A5441, Sigma) antibody was used in 1:1000 concentration for equal loading control. Proteins were visualized using a C-Digit^®^ imaging system (Ll-COR)

## Results and discussion

### Chemistry

**Scheme 1.**
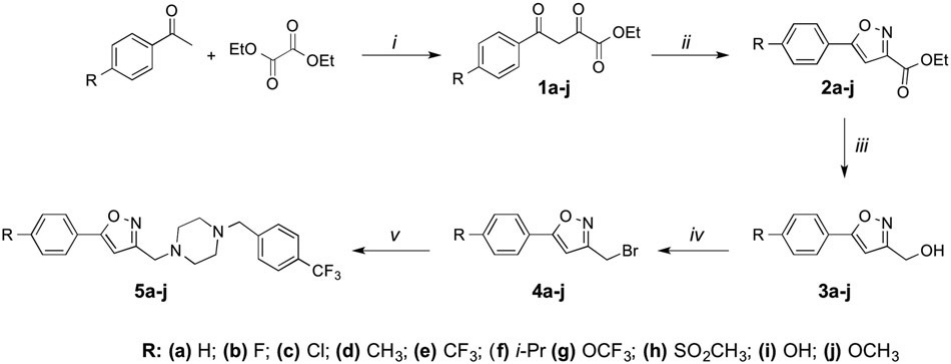
Synthesis of compounds **5a-j**. Reagents and conditions: *i*. NaOEt, EtOH *ii*. NH_2_OH.HCl, EtOH *iii*. LiAlH_4_, THF or NaBH_4_, THF/MeOH *iv*. CBr_4_/PPh_3_, DCM *v*. 4- (trifluoromethyl)benzylpiperazine, DIEA, DMF.

**Scheme 2.**
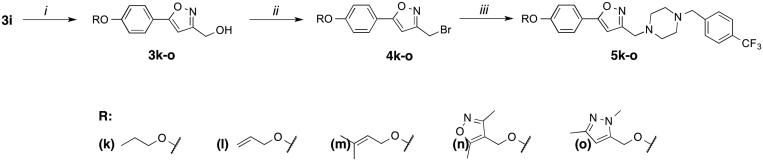
Synthesis of compounds **5k-o**. Reagents and conditions: *i*. R-Br, K_2_CO_3_*ii*. CBr_4_/PPh_3_, DCM *iii*. 4- (trifluoromethyl)benzylpiperazine, DIEA, DMF.

Compounds **5a**–**o** was prepared following the reaction sequence illustrated in Schemes 1 and 2 using the known general methods. Hence, diethyloxalate has been treated with substituted acetophenones in the presence of a base to obtain β-ketoesters **1a**–**j**. These intermediates (**1a**–**j**) were subsequently cyclized with hydroxylamine hydrochloride to provide isoxazole esters **2a**–**j**. Reduction of **2a**–**j** with LAH or NaBH_4_ followed by bromination with CBr_4_/PPh_3_ provided isoxazole methylbromides (**4a**–**j**). Finally, these intermediate alkyl bromides were treated with 4-trifluoromethylbenzylpiperazine to achieve target compounds **5a**–**j**. For the synthesis of compounds **5k**–**o**, alkylation of phenolic hydroxyl of the intermediate **3i** with appropriate alkyl bromides was first accomplished, and then used to produce desired final compounds **5k**–**o** following the reaction sequence shown in Scheme 2. All compounds were purified by automated flash chromatography and checked for purity by TLC and UPLC before being tested in biological assays (purity was 97% based on the peak area percentage of UPLC analysis). The structure of synthesized compounds was confirmed by means of ^1^H NMR, ^13^C NMR and high-resolution mass spectrometry (HRMS).

### Biological evaluation

#### Cytotoxicity of isoxazole-piperazine hybrids 5a–o in liver and breast cancer cells

The newly synthesized compounds (**5a**–**o**) were evaluated for their antitumor activities against human liver (Huh7 and Mahlavu), and breast (MCF7) carcinoma cell lines using the sulforhodamine B (SRB) assay^43^. Data are expressed as IC_50_ values, defined as the half maximal inhibitory concentration, and are shown in Table 1.

**Table 1.** *In vitro* cytotoxic activities of **5a**-**o** with 72 h of treatment.


		IC_50_ (μM)		
Compd No	R	Huh7	Mahlavu	MCF7
**5a**	–H	17.1	14.1	19.9
**5b**	–F	5.3	6.2	11.9
**5c**	–Cl	9.6	26.8	14
**5d**	–CH_3_	24	>40	9.4
**5e**	–CF_3_	19.9	>40	9.5
**5f**	–*i*-Pr	20.8	>40	9.8
**5g**	–OCF_3_	3.6	11.0	10.2
**5h**	–SO_2_CH_3_	4.2	26.0	2.9
**5i**	–OH	3.8	7.5	8.3
**5j**	–OCH_3_	14.6	7.6	14.8
**5k**		8.8	7.7	4.9
**5l**		2.0	1.8	3.5
**5m**		1.3	0.3	2.7
**5n**		1.2	2.8	1.6
**5o**		0.3	3.7	1.2

IC_50_ values were calculated from the cell growth inhibition percentages obtained with five different concentrations in triplicates (*R*^2^ > 0.9).

Among the isoxazole analogues, compound having a non-substituted phenyl attachment at 5-position of the isoxazole nucleus (**5a**) showed the least potent cytotoxic activity for all the three cell lines (IC_50_ = 14.1 – 19.9 µM). However, para fluorine substitution improved the cytotoxic activity, and **5b** appeared to be equally efficient in both liver cancer cells (IC_50_ = 5.3 µM for Huh7 and 6.2 µM for Mahlavu) but was less efficient in breast cancer cells (IC_50_ = 11.9 µM for MCF7), while the chlorine substituted derivative **5c** was 4.3-fold less potent in Mahlavu as compared to **5b** (Table 1). Compounds **5d**–**f** having *p*-alkyl substituents showed only moderate cytotoxic activity against MCF7 breast cancer cells with IC_50_ values of 9.4–9.8 µM but proved to be less potent in liver cancer cells such as Huh7 (IC_50_ values of 19.9–24 µM) and Mahlavu (IC_50_ >40 µM). Introducing polarity to *p*-phenyl (**5g**–**j**) clearly improved the cytotoxic potency for both Huh7 (IC_50_ = 3.6 – 5.4 µM) and MCF7 cells (IC_50_ = 2.9 – 10.2 µM) along with a slight improvement in Mahlavu cells (IC_50_ = 7.5 – 26 µM). Next, O-alkylated analogues of compound **5i** were evaluated for their anti-proliferative activity. As compared to the compounds **5g**–**j**, cytotoxic potency significantly increased by allyl (**5l**), prenyl (**5m**), 3,5-dimethylisoxazol-4-ylmethyl (**5n**) and 1,3-dimethylpyrazol-5-ylmethyl (**5o**) substitutions with IC_50_ values between 0.3 and 3.7 µM, depending on the cell line (Table 1). Based on the promising cytotoxic activities, **5m** and **5o** were selected for further biological studies in order to understand the underlying mechanisms of their anticancer activities.

#### Real-time cellular response of cancer cells upon treatment with compounds 5m and 5o

**Figure 1.**
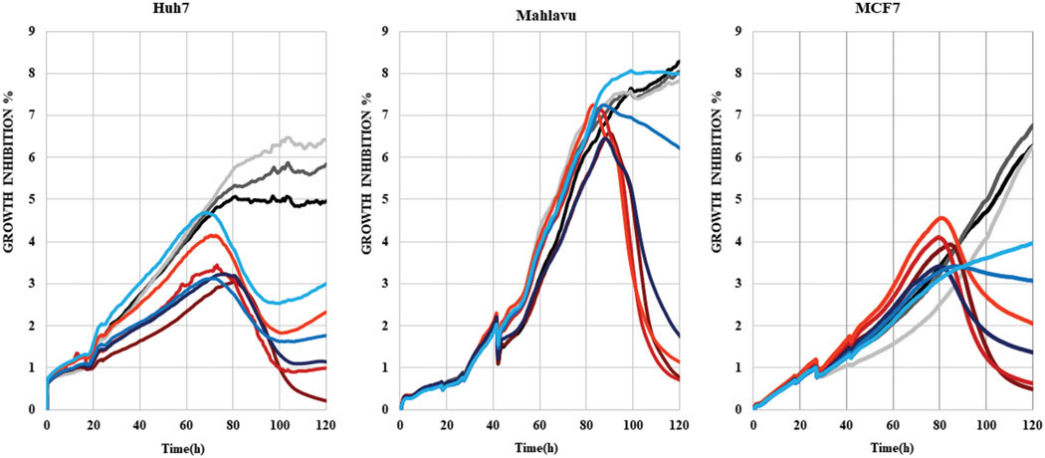
RT-CES analysis of liver cancer cell lines (Huh7 and Mahlavu) and breast cancer cell line (MCF7) treated with compounds **5o** (dark red, red and orange for 10, 5, and 2.5 µM, respectively) and **5m** (dark blue, blue and turquoise for 10, 5 and 2.5 µM, respectively) with DMSO control (0.1%) at different concentrations (black, dark grey and grey for 10, 5 and 2.5 µM, respectively) for 120 h. The experiment was conducted in triplicate and was normalized to DMSO controls.

Time-dependent cytotoxic activities of **5m** and **5o** were scrutinized with real time cell electronic sensing (RT-CES)^44^ by monitoring dynamic cell proliferation of Huh7, Mahlavu, and MCF7 cells. RT-CES assay revealed that **5m**/**5o** significantly reduced the growth rate of cells as compared to DMSO control. This real-time growth pattern confirmed that **5m** and **5o** displayed time and dose-dependent growth inhibitory effects in all cells (Figure 1). Cytotoxic effects of **5m** and **5o** on all three cell lines could be observed after 24 h of compound treatment, and reached to its highest values upon 72 h. As a result of cell cycle arrest or oxidative stress, this real-time growth pattern proposed growth inhibition in which the cells were neither proliferating nor dying, while the DMSO treated cells sustained to increase their number until they reached confluence^45^. In general, the RT-CES results were consistent with values obtained with the SRB assay. In light of this information, the molecular mechanisms underlying the cytotoxic activities of these isoxazole-piperazine hybrids were further investigated in detail with PTEN adequate epithelial like Huh7 cells and PTEN deficient mesenchymal like Mahlavu cells^46^.

#### Oxidative stress induced by compounds 5m and 5o

**Figure 2.**
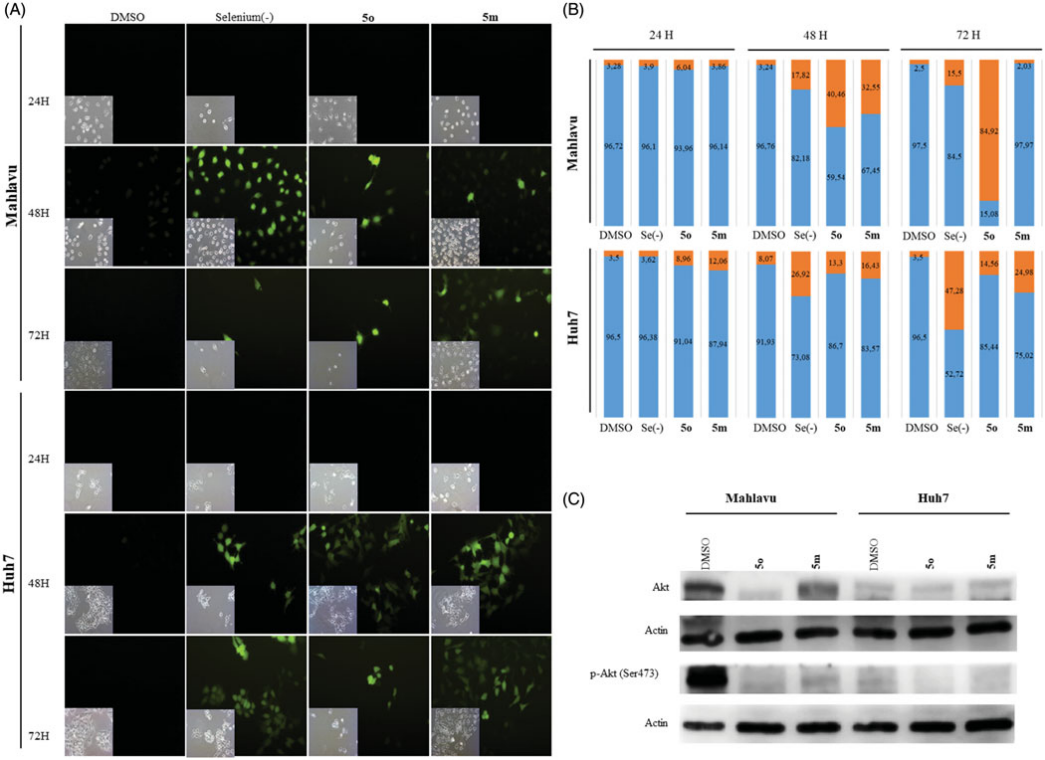
Oxidative stress induced by **5o** and **5m** liver cancer cells, which were treated with **5o** (1 µM for Huh7 and 4 µM for Mahlavu) and **5m** (1 µM for Huh7 and Mahlavu) or DMSO control for 24 h, 48 h, and 72 h. Selenium deficient serum-free medium was used as a positive control of ROS induction. **A.** DCFH-DA staining of the cells under oxidative stress with fluorescent microscope (20×) for 24 h, 48 h, and 72 h. (**B**) Cytometric analysis of oxidative stress induction. ROS positive cells are indicated with orange and ROS negative cells are shown in blue. (**C)** AKT and phospho-AKT in Mahlavu and Huh7 cells treated with **5o** and **5m** for 72h. Actin was used for equal loading.

Reactive oxygen species (ROS), depending on their dose, can alter cellular pathways and promote cell cycle arrest and apoptosis in liver cancer cells^41^. Therefore, induction of oxidative stress by compounds **5m** and **5o** was analyzed in HCC cells (Mahlavu and Huh7) at different time periods (Figure 2). Selenium-deficient serum free medium grown cells were used as experimental positive control for ROS generation^41^. It is shown that while PTEN deficient Mahlavu cells can tolerate selenium deficient serum free medium, PTEN adequate Huh7 cells are strongly affected^41^. For visualization of *in situ* presence of oxidative stress, dichloro-dihydro fluorescein diacetate (DCFH-DA) assay was performed on these cells, which were treated with **5m**/**5o** for 24 h, 48 h and 72 h (Figure 2(A)). In the presence of oxidative stress, DCFH-DA dye was oxidized to a green fluorescent molecule, DCF. Fluorescent microscopy images represented that oxidative stress was triggered by compounds **5m** and **5o**. While compounds **5m** and **5o** started to affect Mahlavu cells after 24 h, **5o** and **5 m** treated Huh7 cells displayed a raise in ROS (+) cells at 24 h (Figure 2(B)), which were in parallel to cell death as determined by RT-CES assay. We illustrated that **5o** leads to an increase in ROS (+) cells with 40% and 13% for 48 h and 85% and 15% for 72 h in Mahlavu and Huh7 cells, respectively, when compared to DMSO controls (Figure 2(B)). In addition, compound **5m** increased ROS (+) cells with 16% for 48 h and 25% for 72 h in Huh7, and it also caused a rise in ROS (+) cells with 32% in Mahlavu cells for 48 h (Figure 2(B)).

Metabolic stress induces cell death through ROS-induced apoptosis and Akt is one of the primary effectors in response to metabolic stress^47^. Akt protein, which is hyperactivated in many tumours, plays a major role in both cell survival and resistance to tumor therapy^48^. While Akt pathway is hyperactive in Mahlavu cells due to PTEN deletion, the pathway is normoactive in PTEN adequate Huh7 cells^49^. Therefore, the poorly differentiated Mahlavu cells are considered as aggressive HCC phenotype. In addition, Akt signaling was illustrated to be involved in the oxidative stress induced cellular pathways^50^. Based on the findings that the compounds **5m** and **5o** caused ROS generation in HCC cells, Akt and phospho(p)-Akt protein levels comparatively analyzed in PTEN deficient Mahlavu and PTEN adequate Huh7 cells (Figure 2(C)). Significant decrease in the levels of Akt and p-Akt proteins was observed in **5m/5o** treated Mahlavu cells as compared to DMSO control. However, compounds treated Huh7 cells did not result in a significant decrease in Akt protein levels. Although Mahlavu cells have hyperactive Akt signal pathway due to the deletion of PTEN gene, which leads to more drug resistant phenotype, more than 80% of Mahlavu cells were ROS positive upon **5o** treatment (Figure 2(B)). Under normal conditions Mahlavu cells exhibit cryptic resistance to intrinsic oxidative stress-induced apoptosis due to selenium deficiency. However, treatment with **5m** and **5o** clearly induces oxidative stress along with cell death that the latter being more effective. Hence, we analyzed the nature of cell death induced with these compounds in liver cancer cells.

#### 5m and 5o induce apoptosis

**Figure 3.**
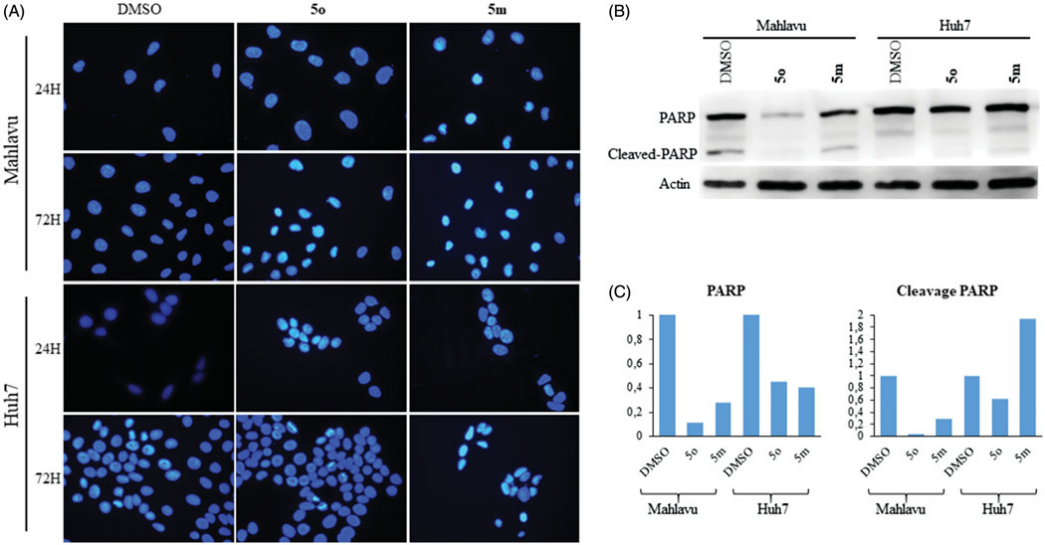
Characterization of cell death using fluorescent microscopy and Western blotting. (**A)** Hoechst staining of **5o** and **5m** treated Mahlavu and Huh7 cells with apoptotic nuclei at 24 h and 72 h. (**B)** PARP in Mahlavu and Huh7 cells treated with **5o** and **5m** for 72 h. Actin was used for equal loading. (**C)** The bar graphs representing relative band intensities of PARP and cleaved-PARP, which were normalized with their actin loading controls.

Compounds **5m** and **5o** induced apoptotic morphological changes as observed by nuclear staining with Hoechst in Huh7 and Mahlavu cells. Following treatment with **5m/5o** for 24 h, 48 h, and 72 h, cells were analyzed under fluorescent microscopy. As shown in Figures 3(A) and S1, typical morphological changes such as chromatin condensation, nuclear fragmentation and apoptotic bodies were detected in treated cells for different time periods.

The activation of apoptotic pathways through **5m** and **5o** treatments were further confirmed with apoptosis-associated PARP protein levels. There was a significant decrease in total PARP protein in **5m**/**5o** treated Mahlavu and Huh7 cells while an increase in PARP cleavage in the **5m** treated Huh7 cells was identified at 72 h. These results supported the increased cytotoxic effects of the compounds on liver cancer cells (Figure 3(B) and 3(C)). Our data indicates that the **5m**/**5o**-induced ROS leading to the cell death characterized with apoptosis. We then analyzed the proteins involved in apoptosis and cell cycle with the aim of further characterization of cell death mechanism.

#### Induction of cell cycle arrest and analysis of cellular pathways targeted by 5m and 5o

**Figure 4.**
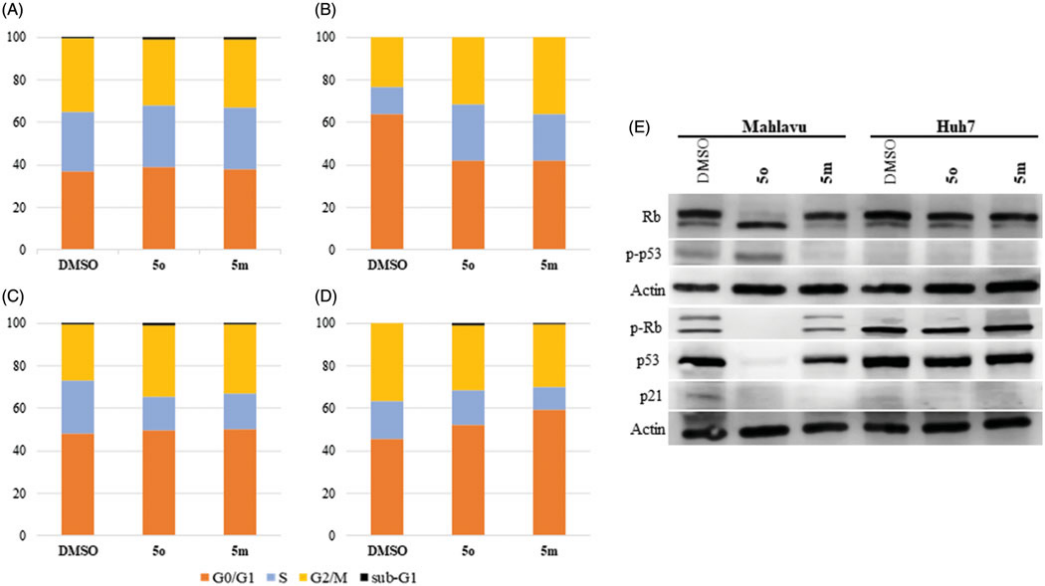
Detection of cell cycle arrest. Cell cycle analysis of Mahlavu for (**A**) 48 h (**B**) 72 h and Huh7 for (**C**) 48 h and (**D**) 72 h after treatment with compounds **5o** and **5m**, and DMSO controls following 48 h and 72 h of treatment. Orange, blue, yellow and black show GO/S1, S, G2/M, and Sub-G1, respectively. (**E**) Rb, p53, phospho-Rb, and phospho-p53 in Mahlavu and Huh7 cells with **5o** and **5m** for 72h. Actin was used for equal loading.

Initially, cell cycle arrest was analysed by flow cytometry analysis using propidium iodide (PI) staining of DNA. Huh7 cells treated with **5m** and **5o** showed an increase in entry to G2/M and G1 phases in 48 h (Figure 4(C)) and 72 h (Figure 4(D)), respectively. While no arrest was observed in Mahlavu cells treated with **5m** and **5o** for 48 h (Figure 4(A)), the cells treated with **5m** and **5o** represented a higher cell population in S and G2/M phases for 72 h (Figure 4(B)). Quantitative results of cell cycle analysis were also revealed in Table S1.

Based on the finding that **5m/5o** caused ROS accumulation, cell cycle arrest and apoptosis, several targets involved in these pathways at the protein level were further investigated by Western blot analyses. Proteins p53, p21, Rb are inhibitory regulators of the cell cycle, and are effectors of responses to cellular stress leading to apoptosis and cell cycle arrest. Since **5m** and **5o** induce cellular stress-associated cell death, we examined the levels of these proteins upon compound treatment. Compound **5o** treated Mahlavu cells exhibited significant alteration in protein levels of Rb, phosphorylated form of Rb (Ser807/811), p53 and phosphorylated form of p53 (Ser15) while p21 protein keeps its expression practically unchanged (Figure 4(E)). The phosphorylated from of p53 tumor-suppressor protein has critical roles for cell cycle arrest in response to DNA injury. It is a known fact that reactive oxygen species damage several complex biomolecues in the cell incuding DNA. Upon DNA damage, p53 is stabilized by phosphorylation and activates the expression of target genes. Primary among these is p21^51^, which provides a direct link between p53 and pRb for cell cycle arrest. The p21 retains pRb in a hypophosphorylated state that inhibits E2F-1 activity and thereby S-phase entry. Our results with **5o** treated Mahlavu cells clearly demonstrate that phosphorylation of p53 is associated with hypophoshorylation of pRb and arest in S phase.

## Conclusion

We synthesized a series of isoxazole-piperazine hybrids and evaluated their cytotoxic activities against human cancer cell lines in comparison to DMSO control. The majority of derivatives showed moderate to significant cytotoxicity in the tested cell lines. As a general conclusion, we observed that the substitution pattern on the phenyl group linked to isoxazole at 5-position has a significant impact on the cytotoxicity and the selectivity of the compounds within the series. Therefore, compounds **5m** and **5o,** the most effective derivatives with respect to antiproliferative activity against hepatocellular cancer cells, were selected for detailed mechanistic studies. By further investigating their molecular effects, we showed that compounds **5m** and **5o** caused generation of ROS, induction of apoptotic cell death, and cell cycle arrest at different phases in HCC cells. Particularly, the bioactivities of **5o** in poorly differentiated aggressive Mahlavu cells were prominent. Mahlavu cells were reported to be resistant to the ROS-induced cell death and drug resistant phenotype^41^. In this study, we clearly demonstated that **5o** induces ROS and inhibits Akt cell survival pathway in Mahlavu cells. Decrease in levels of Akt and phosphorylated form of Akt (Ser473) upon treatment and the status of cell cycle proteins were worth exploring since it provided further information about mechanism of action of the compounds on cancer cells.

In conclusion, we were able to demostrate that our novel compounds induces chemically-induced extrinsic ROS, cell survival pathway inhibition through Akt hyperphosphorylation and apoptosis and cell cycle arrest through p53 protein activation. Future studies may evaluate the detailed cellular networks that are affected by the use of high throughput genomic screening methods such as transcriptome analysis with next generation sequencing in the presence of selected compounds. This may lead to identify molecular targets involved in induction of reactive oxygen species and cell cycle for eventual drug design and development against cancer. Therefore, we think that the synthesis of further derivatives as potent anticancer agents will be promising in terms of elucidating alternative mechanistic effects of isoxazole-piperazine hybrids as well as providing future treatment approaches for HCC associated with obesity non-alcoholic fatty liver disease.

## Supplementary Material

Supplemental Material
