# Supplementary material for: Synthesis and cellular bioactivities of novel isoxazole derivatives incorporating an arylpiperazine moiety as anticancer agents
Source: J Enzyme Inhib Med Chem. 2018 Sep 24;33(1):1352–61. doi: 10.1080/14756366.2018.1504041 (PMC6161610; doi:10.1080/14756366.2018.1504041)
Supplement: Supplemental Material [file IENZ_A_1504041_SM7421.pdf]

Supporting Information

Synthesis and cellular bioactivities of novel isoxazole derivatives incorporating an arylpiperazine moiety as anticancer agents

Burcu Çalışkan[a], Esra Sinoplu[b], Kübra İbiş[a], Ece Akhan Güzelcan[b], Rengül Çetin Atalay[b] and Erden Banoglu[a]

Contents

Synthesis of β-ketoesters (Compounds **1a-j**) 2

Synthesis of Isoxazole esters (Compounds **2a-j**) 3

Synthesis of Alcohols (Compounds **3a-j**) 4

Synthesis of Compounds 3k-o 6

Synthesis of Bromides (Compounds **4a-o)** 7

Figure S1. Hoechst staining of Mahlavu and Huh7 cells with apoptotic nuclei after 48 h. 9

Table S1. Cell cycle analysis of Mahlavu and Huh7 after treatment with compounds **5o** and **5m**, and DMSO controls following 48 h and 72 h of treatment 9

Representative 1H and 13C NMR Spectrum of compound **5j** 10

Synthesis of β-ketoesters (Compounds **1a-j**)

Ethanolic solution of sodium ethoxide was obtained by slowly adding freshly cut metallic sodium (8.4 mmol, 2.1 eq) to absolute ethanol (30 ml) under N_2 atmosphere. To the solution of sodium ethoxide, acetophenone derivative (4 mmol, 1 eq) and diethyloxalate (6 mmol, 1.5 eq) were added and stirred at rt overnight and the resulting precipitate was filtered. The solution of sodium ketoenolate ester in water was acidified with conc. hydrochloric acid to afford a solid, which was filtered, washed with water and dried to give corresponding β-ketoester derivative. (for compound **1i**, 4-hydroxyacetophenone:diethyloxalate: sodium ratio was 1:3:4 eq)

					**HRMS (m/z): [M+H]⁺**	
	**Molecular Formula**	**CAS Registry Number**	**Yield %**	**Melting Point (°C)**	**Calculated**	**Found**
**1a**	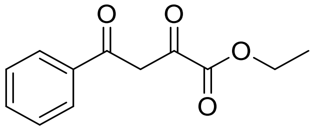	6296-54-4	28.3	165.5-167.0	221.0814	221.0809
**1b**	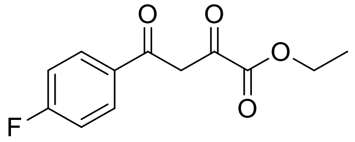	31686-94-9	74.4	48.7-50.4	239.0720	239.0717
**1c**	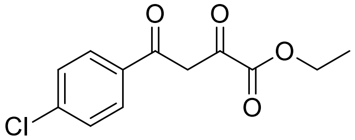	5814-38-0	90.0	61.2-62.5	255.0424	255.0431
**1d**	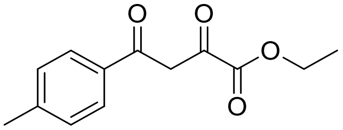	5814-37-9	94.0	40.4-43.2	235.0970	235.0974
**1e**	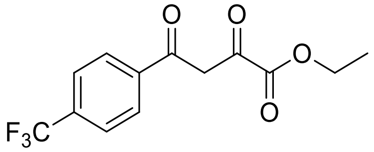	613240-19-0	59.1	56.8-58.0	289.0638	289.0680
**1f**	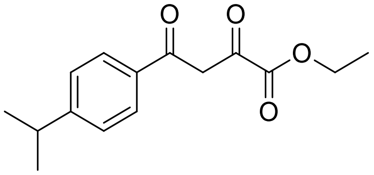	852814-99-4	31.4	164.8-172.3	263.1283	263.1287
**1g**	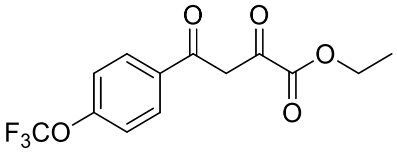	1263284-63-4	63.0	200.5-202.8 (decomp)	305.0637	305.0623
**1h**	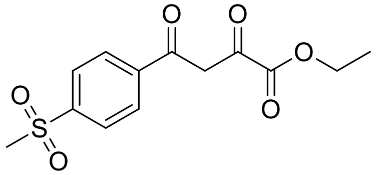	1260599-35-6	77.2	166.5-168.4 (decomp)	299.0589	299.0585

**1i**	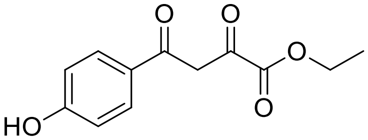	39974-01-1	96.3	148.0-149.7	237.0763	237.0770
**1j**	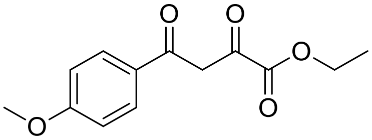	35322-20-4	95.0	54.1-55.3	251.0919	251.0917

Synthesis of Isoxazole esters (Compounds **2a-j**)

To the solution of β-ketoester (2 mmol, 1eq) in ethanol, $NH_2OH.HCl$ (2.5 mmol, 1.25 eq) was added and the resulting mixture was refluxed for 4h. After the completion of the reaction, reaction mixture was cooled and formed cyrstals were collected by filtration and dried. The resulting esters were sufficiently pure by LC-MS analysis for further use.

					**HRMS (m/z): [M+H]⁺**	
	**Molecular Formula**	**CAS Registry Number**	**Yield %**	**Melting Point (°C)**	**Calculated**	**Found**
**2a**	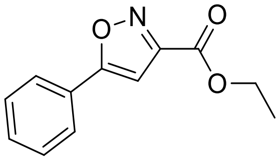	7063-99-2	78.8	48.8-49.1	218.0817	218.0820
**2b**	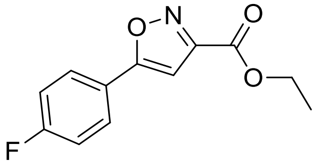	640291-92-5	71.1	112.1-112.9	236.0723	236.0718
**2c**	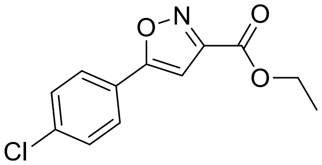	8182-12-4	63.0	125.0-125.7	252.0427	252.0428
**2d**	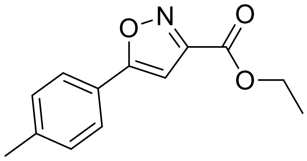	88958-15-0	64.0	57.4-58.7	232.0974	232.0968
**2e**	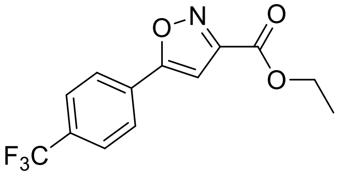	613240-18-9	77.7	134.3-135.0	286.0691	286.0687

**2f**	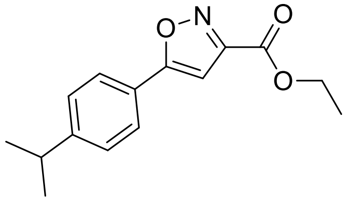	1188158-30-6	80.0	oil	260.1287	260.1289
**2g**	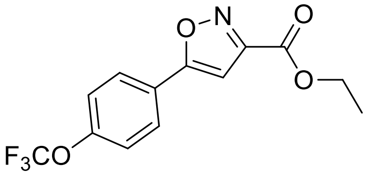	1110771-70-4	83.0	156.7-159.7	302.0640	302.0644
**2h**	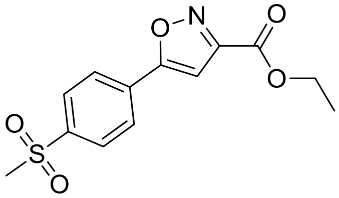	1245062-97-8	74.5	172.1-172.5	296.0593	296.0588
**2i**	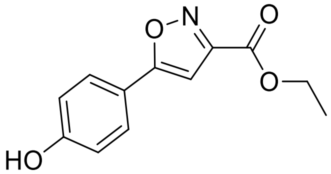	1352896-92-4	89.3	180.3-181.4	234.0766	234.0772
**2j**	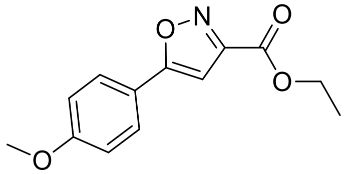	925006-96-8	92.0	89.7-80.1	248.0923	248.0924

Synthesis of Alcohols

Synthesis of Compounds **3a, 3c, 3e, 3f, 3h**

$NaBH_4$ (5.1 mmol, 3 eq) was slowly added to the solution of isoxazole ester (1.7 mmol, 1 eq) in THF:MeOH (2:1) under N_2 atm. The reaction mixture was refluxed for 2h. After the completion of the reaction the mixture was evaporated, water and 2M HCl were added to the residue and formed precipitate was filtered.

Synthesis of Compounds **3b, 3d, 3g, 3i, 3j**

$LiAlH_4$ (3.25 mmol, 1.7 eq) was slowly added to the solution of isoxazole ester (1.91 mmol, 1 eq) in dry THF under N_2 at 0^oC. The reaction mixture was allowed to warm RT and stirred for 5h. The mixture was quenched with 2M HCl and extracted with ethyl acetate. The combined organics were washed with water, brine and dried over Na_2SO_4 and concentrated to provide product.

					HRMS (m/z): [M+H]+	
	**Molecular Formula**	**CAS Registry Number**	**Yield %**	**Melting Point (°C)**	**Calculated**	**Found**
**3a**	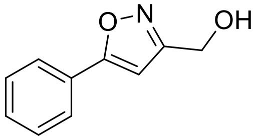	1619-37-0	73.3	101.0-101.3	176.0712	176.0718
**3b**	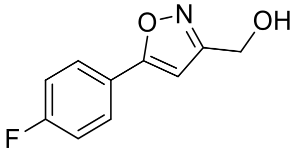	640291-97-0	80.4	80.2-82.8	194.0617	194.0614
**3c**	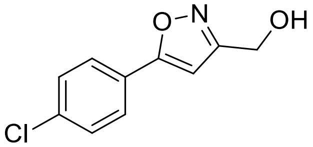	81282-13-5	70.0	96.4-97.1	210.0322	210.0320
**3d**	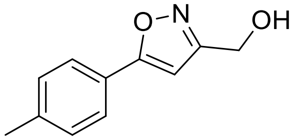	640291-93-6	72.0	112.1-112.9	190.0868	190.0861
**3e**	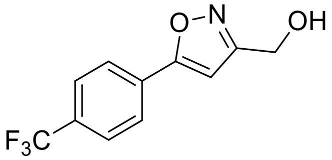	613240-20-3	89.3	115.6-116.9	244.0585	244.0585
**3f**	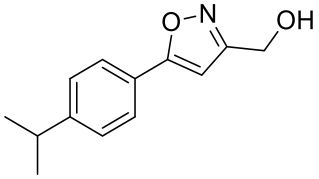	1188158-43-1	48.0	55.3-57.5	218.1181	218.1190
**3g**	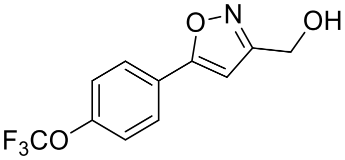	1421959-09-2	66.0	67.6-70.0	260.0535	260.0539
**3h**	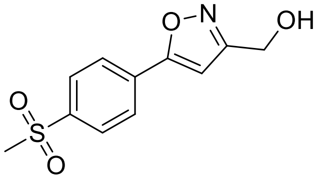	1517560-26-7	87.4	158.4-159.5	254.0487	254.0493
**3i**	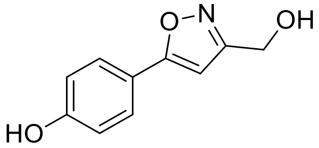	2091378-04-8	90.2	207.7-209.1	192.0661	192.0663

**3j**	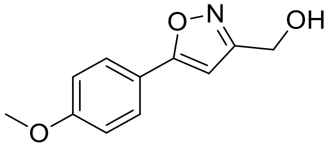	58492-77-6	89.0	89.1-91.1	206.0817	206.0820

Synthesis of Compounds 3k-o

5-(4-propoxyphenyl)isoxazol-3-yl)methanol (**3k**) CAS: 1536438-65-9

The mixture of Compound **3i** (1.05 mmol, 1 eq), 1-bromopropane (3.04 mmol, 2.9 eq) and K_2CO_3 (1.78 mmol, 1.7 eq) in AcCN (4 ml) was heated by microwave irradiation at 120 ^oC for 40 min. Then the reaction mixture was cooled to rt, poured into water, and formed precipitate was filtered. The crude product was purified by preparative LC using water:acetonitril (40%→100%) to give the corresponding product. Yield 53.6 %; mp 94.5-95.8; HRMS (m/z) $[M+H]^+$ calcd for $C_{13}H_{15}NO_3$: 234.1130, found 234.1124.

(5-(4-(Allyloxy)phenyl)isoxazol-3-yl)methanol (**3l**).

The mixture of Compound **3i** (1.1 mmol, 1 eq), allyl bromide (3.3 mmol, 3 eq) and K_2CO_3 (1.43 mmol, 1.3 eq) in acetone (10 ml) was refluxed for 4 h. After completion, inorganic salt was removed by filtration and washed with DCM. The filtrate was evaporated under reduced pressure. The residue was purified by preparative LC using water:acetonitril (40%→100%) to give the corresponding product. Yield 64%; mp 73.3-75.2°C. ^{1}H NMR ($CDCl_3$): δ 2.33 (1H, s), 4.58 (2H, d, J = 5.2 Hz), 4.78 (2H, s), 5.31 (1H, dd, J = 10.4, 0.8 Hz), 5.43 (1H, dd, J = 17.2, 0.8 Hz), 6.01-6.10 (1H, m), 6.44 (1H, s), 6.96 (2H, d, J = 8.4 Hz), 7.68 (2H, d, J = 8.4 Hz). HRMS (m/z) $[M+H]^+$ calcd for $C_{13}H_{14}NO_3$: 232.0974, found, 232.0970.

(5-(4-((3-Methylbut-2-en-1-yl)oxy)phenyl)isoxazol-3-yl)methanol (**3m**).

The mixture of Compound **3i** (1.03 mmol, 1.1eq), 3,3-dimethylallyl bromide (0.94 mmol, 1 eq), K_2CO_3 (1.22 mmol, 1.3 eq) in aceton (10 ml) was stirred at RT for 48h. After the completion of the reaction the mixture was poured into ice-water and formed precipitate collected by filtration. Yield 65.6%; mp 101.1-102.0°C. ^{1}H NMR ($CDCl_3$): δ 1.76 (3H, s), 1.81 (3H, s), 2.08 (1H, bs), 4.56 (2H, d, J = 6.8 Hz), 4.79 (2H, s), 5.47-5.51 (1H, m), 6.44 (1H, s), 6.97 (2H, d, J = 8.8 Hz), 7.69 (2H, d, J = 8.8 Hz). HRMS (m/z) $[M+H]^+$ calcd for $C_{15}H_{18}NO_3$: 260.1287, found, 260.1282.

(5-(4-((3,5-Dimethylisoxazol-4-yl)methoxy)phenyl)isoxazol-3-yl)methanol (**3n**).

The mixture of Compound **3i** (1.31 mmol, 1 eq), 4-chloromethyl-3,5-dimethylisoxazole (1.57 mmol, 1.2 eq) and K_2CO_3 (2.62 mmol, 2 eq) in DMF (3 ml) was heated by microwave irradiation at 120 ^oC for 20 min. Then the reaction mixture was poured into ice-water and formed precipitate was filtrated. The crude product was purified by preparative LC using water:acetonitril (45%→100%) to give the corresponding product. Yield 52.2%; mp 132.7-134.0°C. ^{1}H NMR ($CDCl_3$): δ 2.29 (3H, s), 2.42 (3H, s), 4.80 (2H, s), 4.83 (2H, s), 6.48 (1H, s), 7.01 (2H, d, J = 8.6 Hz), 7.73 (2H, d, J = 8.6 Hz). HRMS (m/z) $[M+H]^+$ calcd for $C_{16}H_{17}N_2O_4$: 301.1188, found, 301.1193.

(5-(4-((1,3-Dimethyl-1H-pyrazol-5-yl)methoxy)phenyl)isoxazol-3-yl)methanol (**3o**).

Compound **3i** (1.1 mmol, 1 eq), 5-chloromethyl-1,3-dimethy-1H-pyrazol (1.43 mmol, 1.3 eq) and K_2CO_3 (3.3 mmol, 3 eq) in DMF (3 ml) was heated by microwave irradiation at 120 ºC for 20 min. Then the reaction mixture was poured into ice-water and formed precipitate was filtrated. The crude product was purified by preparative LC using water:acetonitril (45%→100%) to give the corresponding product. Yield 60.3%; mp 162.0-163.0 °C. ^{1}H NMR ($CDCl_3$): δ 2.25 (3H, s), 3.85 (3H, s), 4.79 (2H, s), 5.03 (2H, s), 6.12 (1H, s), 6.48 (1H, s), 7.03 (2H, d, J = 8.8 Hz), 7.72 (2H, d, J = 8.8 Hz). HRMS (m/z) $[M+H]^+$ calcd for $C_{16}H_{18}N_3O_3$: 300.1348, found, 300.1344.

Synthesis of Bromides (Compounds **4a-o)**

To a solution of starting material alcohol (**3a-o**) (1.34 mmol, 1eq) in DCM, CBr_4 (1.61 mmol, 1.2 eq) and PPh_3 (2.01 mmol, 1.5 eq) were added at 0ºC and the mixture was stirred for 3 h. Upon completion, the reaction mixture was diluted with DCM, organic phase washed with water, dried over Na_2SO_4, and concentrated under reduced pressure. The resulting crude product was purified by flash chromatography using hexane:ethyl acetate.

					**HRMS (m/z): [M+H]⁺**	
	**Molecular Formula**	**CAS Registry Number**	**Yield %**	**Melting Point (˚C)**	**Calculated**	**Found**
**4a**	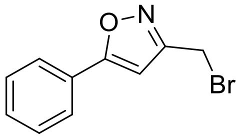	154016-50-9	95.0	64.9-65.5	237.9868	237.9869
**4b**	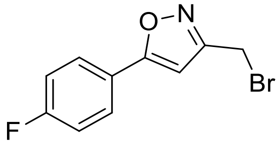	1267329-49-6	94.5	72.4-74.2	255.9773	255.9782
**4c**	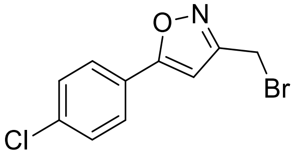	323594-75-8	94.3	97.4-98.2	271.9478	271.9477
**4d**	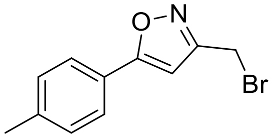	1267605-54-8	83.6	83.0-85.3	252.0024	252.0020
**4e**	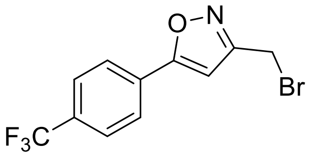	952710-03-1	93.0	74.2-75.4	305.9741	305.9744

**4f**	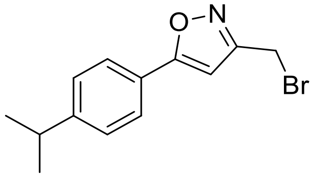	1266892-79-8	93.0	oil	280.0337	280.0329
**4g**	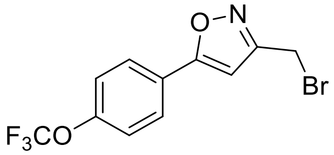	1421959-10-5	89.0	38.2-41.1	321.9690	321.9678
**4h**	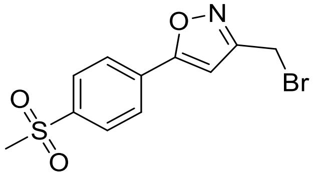	1503711-59-8	87.0	144.3-145.0	315.9643	315.9648
**4j**	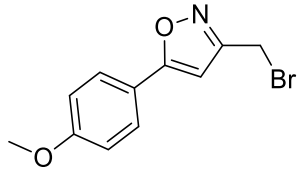	934603-49-3	90.8	80.7-81.9	267.9973	267.9964
**4k**	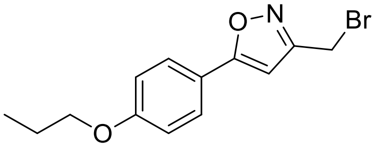	1528534-49-7	90.5	61.7-62.7	296.0286	296.0284

4-(3-(Bromomethyl)isoxazol-5-yl)phenol (**4i**).
Yield 45.0%; mp 149.5-151.1 °C. ^{1}H NMR ($CDCl_3$): δ 4.44 (2H, s), 5.26 (1H, bs), 6.48 (1H, s), 6.92 (2H, d, J = 8.8 Hz), 7.67 (2H, d, J = 8.8 Hz). HRMS (m/z) $[M+H]^+$ calcd for $C_{10}H_9BrNO_2$: 253.9817, found, 253.9813.

5-(4-(Allyloxy)phenyl)-3-(bromomethyl)isoxazole (**4l**).
Yield 80.5%; mp 50.0-50.7°C. ^{1}H NMR ($CDCl_3$): δ 4.45 (2H, s), 4.58-4.60 (2H, m), 5.32 (1H, dq, J = 10.4, 1.4 Hz), 5.43 (1H, dq, J = 17.2, 1.6 Hz), 6.01-6.11 (1H, m), 6.48 (1H, s), 6.97 (2H, d, J = 9.2 Hz), 7.70 (2H, d, J = 9.2 Hz). HRMS (m/z) $[M+H]^+$ calcd for $C_{13}H_{13}BrNO_2$: 294.0130, found, 294.0127.

3-(Bromomethyl)-5-(4-((3-methylbut-2-en-1-yl)oxy)phenyl)isoxazole (**4m**)
Yield 58.3%; mp 79.3-80.4°C. ^{1}H NMR ($CDCl_3$): δ 1.76 (3H, s), 1.81 (3H, s), 4.44 (2H, s), 4.56 (2H, d, J = 6.8 Hz), 5.47-5.51 (1H, m), 6.47 (1H, s), 6.98 (2H, d, J = 9.0 Hz), 7.69 (2H, d, J = 9.0 Hz). HRMS (m/z) $[M+H]^+$ calcd for $C_{15}H_{17}BrNO_2$: 322.0443, found, 322.0447.

4-((4-(3-(Bromomethyl)isoxazol-5-yl)phenoxy)methyl)-3,5-dimethylisoxazole (**4n**).
Yield 91.0%; mp 107.8-109.5°C. ^{1}H NMR ($CDCl_3$): δ 2.30 (3H, s), 2.42 (3H, s), 4.45 (2H, s), 4.84 (2H, s), 6.51 (1H, s), 7.02 (2H, d, J = 9.0 Hz), 7.73 (2H, d, J = 9.0 Hz). HRMS (m/z) $[M+H]^+$ calcd for $C_{16}H_{16}BrN_2O_3$: 363.0344, found, 363.0341.

3-(Bromomethyl)-5-(4-((1,3-dimethyl-1H-pyrazol-5-yl)methoxy)phenyl)isoxazole (**4o**).
Yield 84.5%; mp 132.2-133.4°C. ^{1}H NMR ($CDCl_3$): δ 2.26 (3H, s), 3.85 (3H, s), 4.44 (2H, s), 5.04 (2H, s), 6.12 (1H, s), 6.50 (1H, s), 7.04 (2H, d, J = 8.8 Hz), 7.72 (2H, d, J = 8.8 Hz). HRMS (m/z) $[M+H]^+$ calcd for $C_{16}H_{17}BrN_3O_2$: 362.0504, found, 362.0505.

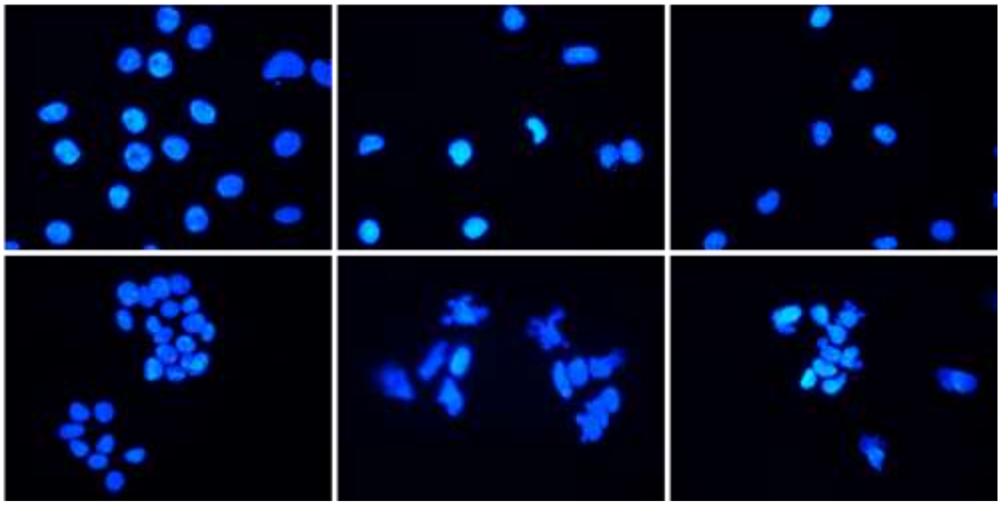

Figure S1. Hoechst staining of Mahlavu and Huh7 cells with apoptotic nuclei after 48 h.

Table S1. Cell cycle analysis of Mahlavu and Huh7 after treatment with compounds **5o** and **5m**, and DMSO controls following 48 h and 72 h of treatment.

**MV (48 h)**	**G0/G1**	**S**	**G2/M**	**Sub-G1**
**DMSO**	37.1	27.7	34.7	0.5
**5o**	39.2	28.7	31.3	0.8
**5m**	38.2	28.8	32.3	0.8
**MV (72 h)**				
**DMSO**	63.8	12.9	23.2	0.1
**5o**	41.9	26.6	31.4	0.1
**5m**	41.8	22.3	35.9	0
**Huh7 (48 h)**				
**DMSO**	48.3	25	26	0.7
**5o**	49.5	15.9	33.8	0.8
**5m**	50	17.1	32.2	0.7
**Huh7 (72 h)**				
**DMSO**	45.7	17.7	36.45	0.15
**5o**	52.3	16.45	30.5	0.75
**5m**	59.3	10.5	29.65	0.55

Representative 1H and 13C NMR Spectrum of compound 5j

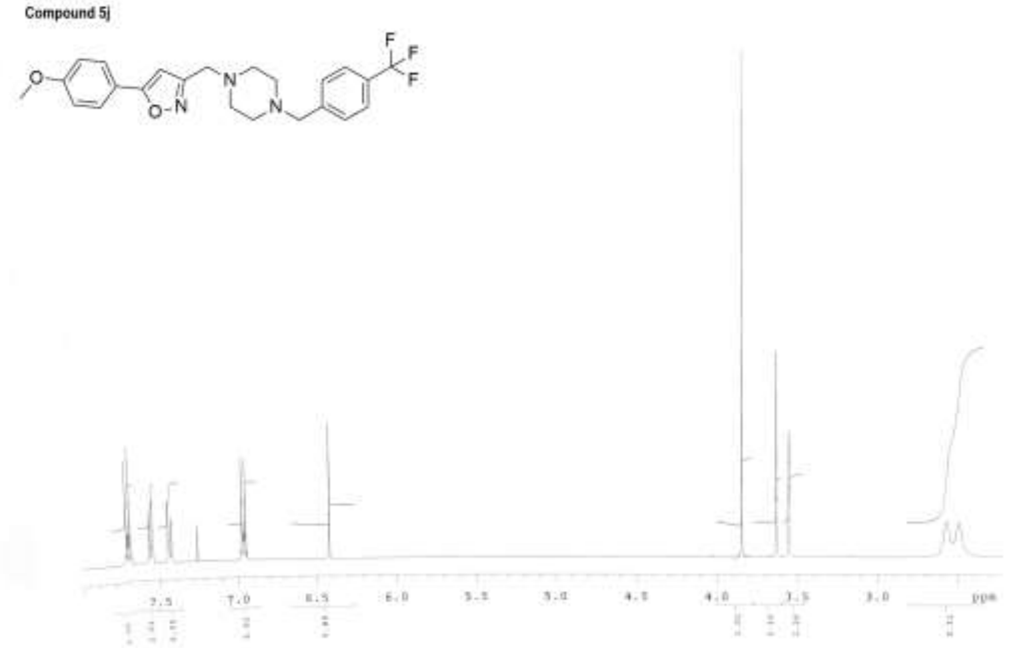

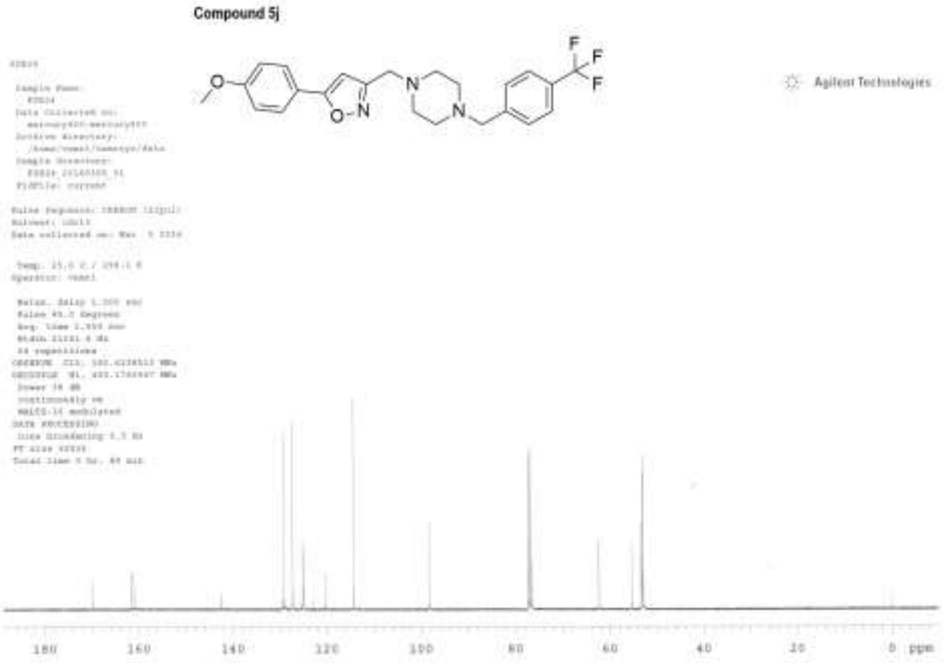

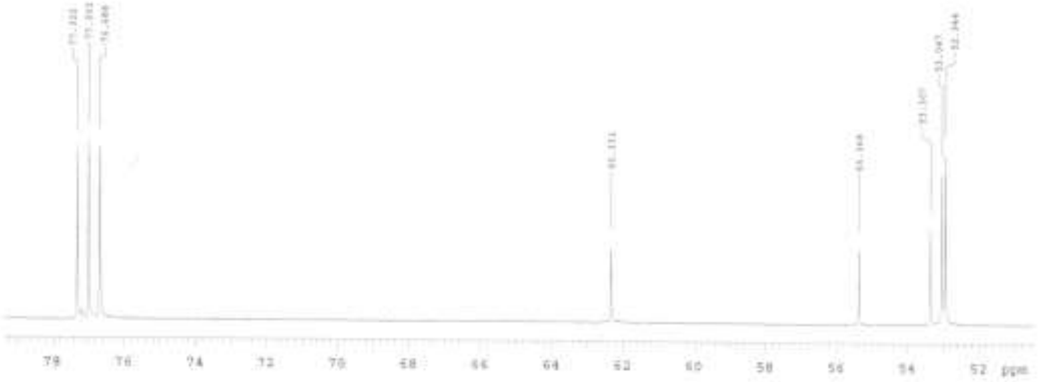
78 76 74 72 70 68 66 64 62 60 58 56 54 52 ppm

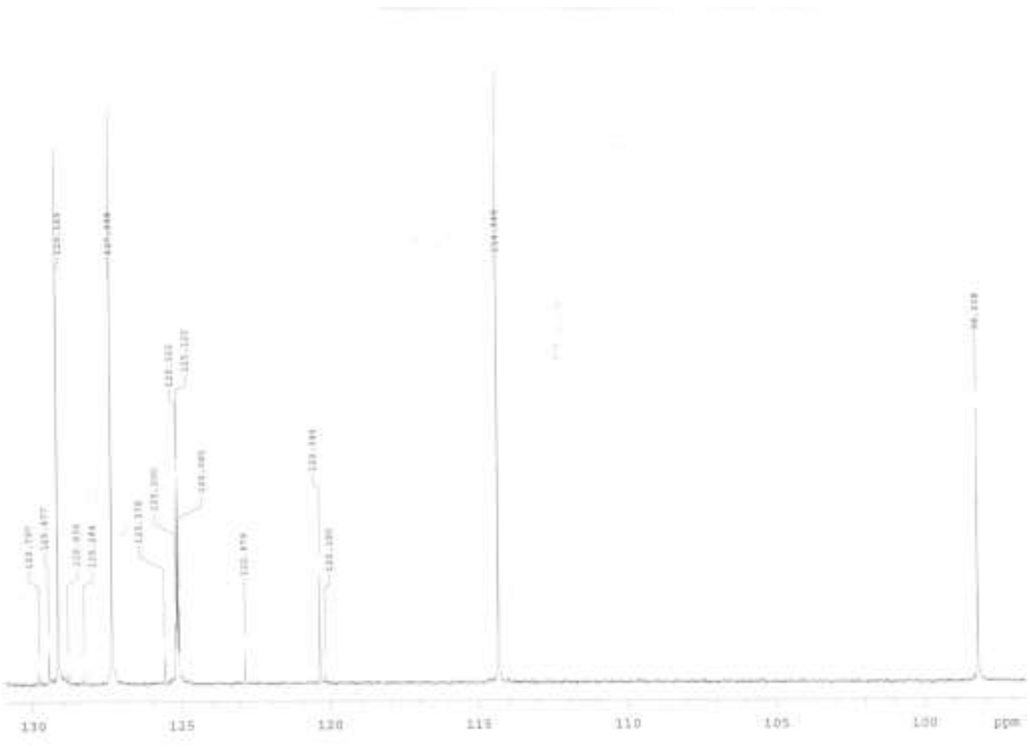
130 125 120 115 110 105 100 ppm

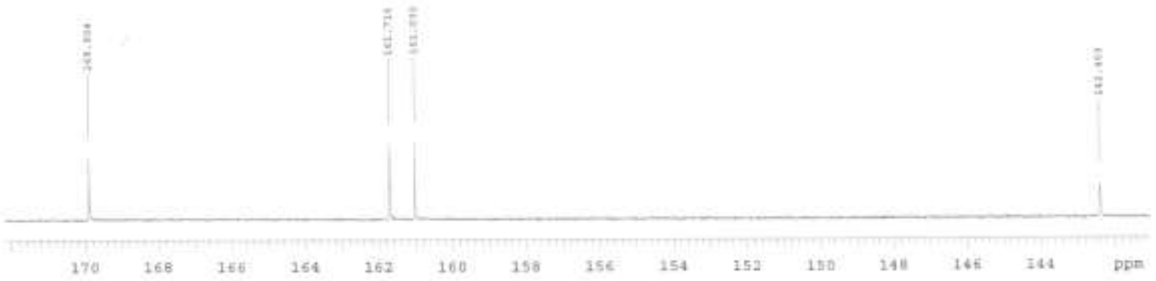
170
168
166
164
162
160
158
156
154
152
150
148
146
144
ppm
